# Lithium use in special populations

**DOI:** 10.4103/0019-5545.37325

**Published:** 2007

**Authors:** E. Mohandas, V. Rajmohan

**Affiliations:** Department of Psychiatry, Elite Mission Hospital, Trichur, Kerala, India

Lithium, a monovalent cation, was first used for the treatment of mania in the 1940s. Half a century into its use, the drug continues to be the preeminent choice for bipolar disorder with antimanic, antidepressant (modest) and antisuicidal property. Lithium is the “gold standard” mood stabilizer against which potential mood stabilizer agents are judged. The therapeutic uses of lithium also include use as an augmenting agent in depression, schizoaffective disorder, aggression, impulse control disorder, eating disorders, attention deficit disorder and in certain subsets of alcoholism. Lithium has been used in many medical disorders, especially cluster headache and dermatological disorders (seborrheic dermatitis, eczematoid dermatitis, genital herpes).[1] The drug is however associated with neurologic, endocrine, cardiovascular, renal, dermatologic and gastrointestinal adverse effects and possible teratogenicity.

## HISTORY

Lithium was first discovered and defined by Johan August Arfvedson in 1817 when he did an analysis of the mineral petalite [LiAl(Si2O5)2]. Petalite was first found by Brazilian scientist Josá Bonifécio Andrade e Silva in 1800. Lepidolite, spodumene, petalite and amblygonite are the more important minerals containing lithium. It was Arfwedson's laboratory chief John Jacob Berzelius who named this alkali metal “lithion.” Arfvedson was never able to fully isolate lithium, and it wasn't until 1855 that it was isolated by William Thomas Brande. Brande and Sir Humphrey Davy earlier had done electrolysis on lithium oxide in 1818. Lithium was first produced commercially in 1923 by Metallgesellschaft AG.[2]

The use of lithium for medicinal purposes can be traced back 1,800 years to the Greek physician Galen, who treated patients with mania by having them bathe in alkaline springs and drink the water, which probably contained lithium. In 1843 Alexander Ure introduced lithium into modern medicine, and he showed the *in vitro* reduction of weight of a uric acid bladder stone in a lithium carbonate solution. Sir Alfred Garrod later discovered that gouty uric acid deposits also were soluble in lithium solution. The view in that time was that uric acid imbalances caused a wide range of diseases, and Armand Trousseau and Alexander Haig proposed that mania and depression also may result from this imbalance and lithium may be effective in these conditions. In the 1840s, lithium was mixed with carbonate or citrate to form a salt and was used to treat gout, epilepsy, diabetes, cancer and insomnia. In the 1870s, the then American Surgeon General William Hammond had provided anecdotal evidence for the use of lithium bromide in the treatment of acute mania. In the 1880s and 1890s the Lange brothers Carl and Fritz used lithium in depression, and Carl Lange was the first to systematically use lithium in the acute and prophylactic treatment of depression.[2] The introduction of lithium preparations and tablets in the 1900s brought to the fore the toxic effects of the drug; and there were reports of weakness, tremor, diarrhea, vomiting and deaths. The drug disappeared from the British Pharmacopoeia by 1932, but later in the 1940s it was used as a sodium substitute in low sodium diets; but the reports of severe intoxication led to its removal from American markets in 1949.[1]

The appearance in 1949 in the *Medical Journal of Australia* of a paper entitled “Lithium salts in the treatment of psychotic excitement” by John F. J. Cade was an unspectacular entry into a new era of psychiatry. Manic patients showed improvement, with the patient becoming calmer after four to five days. There was no improvement in the excited schizophrenic patients, though there was a calming effect. There was no improvement or deepening of depression. The paper also gave details of initial dosage, maintenance doses, appearance of toxic symptoms and warning about lithium over-dosage.[3] Most of the subsequent evidence on lithium was gathered by the European trials, especially by Mogens Abelin Schou from Denmark.[4] The earliest report of lithium treatment in North America was published in 1960. Between 1950 and 1974, 782 papers were published on lithium from Europe, 353 papers from North America and 95 papers from other continents; and this led to the establishment of lithium as an efficacious and well-tolerated drug in mania. The clinical significance of lithium was recognized in a special section of the American Journal of Psychiatry in 1968. In 1970 it was approved by the United States Food and Drug Administration (USFDA) for the treatment of mania, and in 1974 it was approved for maintenance therapy of patients with mania [Table 1].[5]

**Table 1:** Landmarks in the history of lithium

Year	Landmark
1817	Johan August Arfvedson discovers lithium
1843	Alexander Ure introduces lithium in modern medicine
1855	William Thomas Brande fully isolates lithium
1870s	William Hammond - anecdotal evidence of lithium bromide in treatment of acute mania
1890s	Carl Lange - systematic use of lithium in the acute and prophylactic treatment of depression
1900s	Toxicity reports – weakness, tremor, diarrhea, vomiting and deaths
1932	Lithium disappears from British Pharmacopoeia
1940s	Use as sodium substitute in low-sodium diets
1949	Removal from American markets following reports of severe intoxication
1949	John F. J. Cade - use of lithium in acute mania
1950 to 1974	Intense clinical research into safety and efficacy of lithium
1968	American Journal of Psychiatry recognizes the clinical significance of lithium
1970	USFDA approval for treatment of mania
1974	USFDA approval for maintenance therapy of patients with mania

## LITHIUM USE AND CARDIOVASCULAR DISEASES

Lithium is shown to produce a variety of cardiovascular effects in man and experimental animals. These effects are more profound during lithium intoxication, though they can occur at therapeutic levels of lithium. These effects include hypotension, bradycardia (acute effects more common, though profound bradycardia as a late consequence of chronic lithium poisoning has also been reported),[6] decreased cardiac output, cardiac arrhythmias (heart blocks and bradyarrhythmias, especially during intoxication) and possible antiarrhythmic action against experimentally induced arrhythmias.[7] Lithium, however, does not have clinically significant effect on blood pressure. Lithium may also induce various electrocardiographic (ECG) changes, including nonspecific T-wave flattening, dysfunction of sinus node, atrioventricular conduction disturbances and reversible premature ventricular contractions. However, the effect of lithium on QT interval has not been fully elucidated.[8] At therapeutic concentration, T-wave flattening is seen in 30–100% of lithium-treated population. Depressed sinus node function was significantly more common in a lithium-treated population than in an age-stratified reference group. Clinically significant dysfunction, however, seems to be uncommon, with a prevalence of only 0–46% of lithium treatment in a pacemaker population. Mechanism by which lithium depresses sinus node function is not fully understood. Animal experiments indicate that lithium depresses the intracellular potassium (IK) concentration. In addition, lithium replaces intracellular calcium (ICa). Interaction between lithium and IK, ICa, the sodium/ calcium (Na/ Ca) exchange currents and sodium/ potassium (N/ K) pump have been suggested. These disturbances seem to induce various electrophysiological changes, including a decrease of the depolarization rate and reduced electrical impulse propagation.[9] Lithium reduces the mobilization of calcium ion from intracellular pools by inositol triphosphate (IP3)-dependent calcium channels. Lithium inhibits the G protein transduction mechanism linked to type I cholinergic receptors and blocks inositol monophosphatase. Moreover, lithium reduces the production of cyclic adenosine monophosphate (cAMP) and inhibits the influx of calcium ion by limiting its channel opening, and these may interfere with SA and AV node function.[10] There is also evidence that hypotensive and cardiac-depressant effects of lithium chloride are mediated by activation of adenosine triphosphate-sensitive potassium channels.[7] It has also been suggested that lithium might decrease the sensitivity of the sinus node to sympathetic stimulation.[9] In experimental studies, lithium has been shown to enter cardiac cells, displace cations and result in intracellular metabolic changes; including intracellular potassium depletion, which may be one of the mechanisms resulting in T-wave changes on ECG. Calcium channel blockers (especially verapamil) and beta blockers have a synergistic effect with lithium on the severity of bradycardia. SA and AV nodes depend to a large extent on calcium influx for action potentials that maintain their automaticity, and its suppression by calcium channel blockers causes sinus bradycardia and prolongs AV conduction time. Both beta blockers and lithium reduce the production of the second messenger, cAMP, and in turn inhibit the opening of the calcium ion influx.[10]

The drugs that impair renal function, like angiotensin-converting enzyme (ACE) inhibitors, angiotensin II receptor antagionists and certain diuretics, might predispose to lithium toxicity with resultant cardiac manifestations.[1] Conversely, bronchodilators can increase lithium excretion and reduce lithium levels and trigger a relapse.[11]

A patient on lithium who develops myocardial infarction may be treated by temporary lithium discontinuation or by lithium continuation with frequent blood-level monitoring in cases where there is a high chance of relapse. Lithium patient can undergo coronary artery bypass graft (CABG) safely under close supervision. In such cases, lithium should be stopped prior to surgery and restarted at lower dose with serum lithium monitoring. Lithium is known to exacerbate or ameliorate congestive cardiac failure; and in all cases where it exacerbates CCF, lithium may be discontinued. Attention should be paid to hydration status, electrolyte balance and drug interactions in patients with cardiac complications [Table 2].[11]

**Table 2:** Cardiovascular effects of lithium

Hypotension
Bradycardia
Decreased cardiac output
Cardiac arrhythmias (heart blocks and bradyarrhythmias)
Possible antiarrhythmic action
Clinically insignificant effect on blood pressure
Nonspecific T-wave flattening
Dysfunction of sinus node
Atrioventricular conduction disturbances
Reversible premature ventricular contractions
? QT interval changes

## LITHIUM USE AND ENDOCRINE DISORDERS

Lithium is associated with a 7% (2-15%) increase of clinical hypothyroidism, 5% risk of goiter and rarely (0.7%) hyperthyroidism. Subclinical hypothyroidism (approximately 19%) is considered more common than clinical hypothyroidism, and minor elevation of thyroid stimulating hormone (TSH) may normalize without treatment. Chemical hypothyroidism with lithium is around 50%. Lithium is highly concentrated in the thyroid gland against a concentration gradient, probably by active transport. Lithium interferes with glandular release of thyroid hormones (T4 and T3) by decreasing the endocytosis of thyroid hormone-laden thyroglobulin on the luminal side of the thyroid follicle; this causes a transient thyrotropin elevation in more than a third of lithium carbonate-treated patients.[12] The glandular release inhibition is mediated by cyclic adenosine monophosphate (cAMP) within the thyrocyte.[13] Lithium at higher doses may block iodine uptake and organification within the thyroid. Lithium was found to stimulate cell proliferation in the absence of thyrotropin stimulation; but under thyrotropin stimulation, lithium diminished thyrocyte proliferation, especially when used at higher concentrations.[14] Lithium affects many aspects of cellular and humoral immunity *in vitro* and *in vivo.* Prevalence of specific thyroid antibodies among lithium-treated patients varies across studies. Women are known to express thyroid autoimmunity more frequently than men, and it is more in the middle age range. So also thyroid autoimmunity has been found associated with affective disorders, irrespective of lithium use. So it is unclear as to whether lithium *per se* can induce thyroid autoimmunity.[15]

There is evidence that females, patients with rapid cycling and patients with an underlying autoimmune thyroiditis are more prone to lithium-induced hypothyroidism.[16] A study showed that 74% cases of hypothyroidism developed in the first two years of treatment.[14] Lithium-induced goiter is usually characterized by small, smooth and nontender nodules; in some cases, nodules may regress over time. The cause of lithium-induced thyrotoxicosis is not clear; some authorities have speculated that lithium may directly stimulate autoimmune reactions.[14]

It is suggested that before starting lithium, thyroid functions have to be assessed (the determination of thyroid hormones, thyroid stimulating hormone (TSH) and baseline antithyroid antibody). Subsequently, monitoring of thyroid function is done every 6 to 12 months. It is suggested that age and gender should be taken into account while testing for thyroid abnormalities in lithium-treated patients. The testing therefore might have to be revised to include more frequent testing for females over the age of 45 or 50 (every 3 months); while men and young patients could have less frequent tests (every 6 or 12 months).[15] There is still no agreement as to whether lithium treatment poses the risk of aggravating a preexisting adequately treated hypothyroidism. Lithium, however, can be given to these patients under strict monitoring of thyroid function and appropriate dosage adjustment of exogenous thyroid hormone.[11]

Subclinical increase of the levels of calcium and parathormone (PTH) are reported in lithium-treated patients. Very rare reports have been there of lithium-associated hypercalcemia and hyperparathyroidism. In all patients with preexisting hyperparathyroidism, routine monitoring of serum calcium should be performed when they are exposed to lithium. If there is evidence of symptomatic hypercalcemia during lithium treatment, lithium should be discontinued.[11]

There is evidence of increased, decreased and unchanged glucose tolerance while on lithium treatment. There is evidence that lithium has effect on glucose metabolism and has the ability to increase the release of glucagon. Studies have suggested that lithium treatment may impair glucose tolerance or produce frank diabetes in certain patients, and the risk is higher in patients above the age of 40 years. Periodic blood glucose monitoring is recommended in this group of patients.[11] There is evidence that glycosuria associated with hyperglycemia induces osmotic diuresis. Osmotic diuresis increases the renal clearance of lithium, necessitating higher lithium doses to maintain therapeutic lithium plasma concentrations [Table 3].[11]

**Table 3:** Endocrine effects of lithium

Clinical hypothyroidism - 2 to 15%
Subclinical hypothyroidism - approximately 19%
Chemical hypothyroidism - 50%
Goiter - 5%
Hyperthyroidism - 0.7%
Lithium interferes with glandular release of thyroid hormones
Lithium at higher doses blocks iodine uptake and organification
? Lithium-induced thyroid autoimmunity
Subclinical increase of the levels of calcium and PTH
Very rarely, hypercalcemia and hyperparathyroidism
Increased, decreased and unchanged glucose tolerance
Thyroid function test (TFT) every 6 to 12 months
Females over the age of 45 or 50 - every 3 months

## LITHIUM USE AND RENAL DISEASES

The debate regarding the potential nephrotoxic effect of lithium is far from over despite extensive research regarding the same. Polyuria, nocturia and polydipsia occur in approximately 70% of lithium-treated patients. The presence of nephrogenic diabetes insipidus is around 12-20%. Lithium treatment reduces renal concentrating ability by 7-10% and raises the urine volume by 10-20%. Very rarely, nephrotic syndrome occurs as part of lithium treatment. Current evidence suggests that there is no increase in glomerular filtration rate (GFR) even after years of lithium therapy.[11] Histological changes, however, have been reported in renal biopsy specimens of lithium-treated patients, though they cannot be clinically correlated in terms of GFR and chronic lithium use.[17]

Lithium is freely filtered by the glomerulus, and around 80% of it is reabsorbed in the proximal tubule while the other 20% is reabsorbed between the loop of Henle and the collecting duct. The amiloride-sensitive sodium channel and the sodium-proton exchanger serve as the major lithium transporters. Factors which decrease GFR and increase proximal tubular reabsorption (especially volume depletion) will cause raised serum lithium levels. On the other hand, carbonic anhydrase inhibitors, aminophylline and osmotic diuretics decrease proximal tubule sodium reabsorption and increase lithium excretion.

Nephrogenic diabetes insipidus (NDI) and polyuria are due to the inhibitory effects of lithium on cAMP-dependent action of antidiuretic hormone (ADH) on distal tubules and collecting duct. Lithium interferes with the cAMP by its G-proteins antagonizing action. Management strategies for NDI and polyuria include dose reduction, single daily dosing, potassium supplementation, use of amiloride (which blocks the entry of lithium to ADH-sensitive epithelia and enhances ADH action) or hydrochlorothiazide, use of desmopressin and use of indomethacin (as high levels of PGE2 have been found in NDI).[11]

There is hardly any data regarding lithium use in renal disease, and there is a possibility that impairment in renal function may result in decreased lithium clearance and intoxication. The risk of lithium intoxication is higher in patients with renal conditions producing acidosis or urinary acidification defects.[18] Lithium is absolutely contraindicated in acute renal failure but can be used with caution in patients with chronic renal failure.[11] Lithium has also been used in a small number of hemodialysis patients. It is suggested that if essential, lithium should be administered either in the dialysate or as a single dose following each dialysis.[18] Lithium has also been used in patients with renal transplant, and results are more satisfactory in living related donor allograft recipients than cadaveric allograft. Cyclosporine used as immunosuppressant in transplant patients reduces lithium excretion.[19]

Regular monitoring of renal function is therefore necessary during lithium prophylaxis, and there is no optimal monitoring schedule. Recommendations on how frequently serum creatinine levels should be monitored range from every three months to one year. The other tests recommended are urinalysis; clinical estimate of urine volume; and in certain cases, 24-hour urine volume, protein and creatinine clearance [Table 4].[19]

**Table 4:** Renal effects of lithium

Polyuria, nocturia and polydipsia – 70%
Nephrogenic diabetes insipidus – 12 to 20%
Reduced renal concentrating ability by 7 to 10%
Raises the urine volume by 10 to 20%
Very rarely, nephrotic syndrome
No increase in glomerular nitration rate (GFR)
Histological changes
Freely filtered by the glomerulus
80% reabsorbed in the proximal tubule
20% reabsorbed between the loop of Henle and the collecting duct ·↓ GFR and ↑ proximal tubular reabsorption -↑ serum lithium levels
Lithium intoxication ↑ in acidosis or urinary acidification defects
Inhibitory cAMP-dependent action of ADH causing NDI
Cautious use in hemodialysis and transplant cases
Absolutely contraindicated in acute renal failure
Cautious use in chronic renal failure
Serum creatinine levels monitoring (every three months or one year)

## LITHIUM USE AND DERMATOLOGICAL DISEASES

Cutaneous side effects of lithium were first described by Callaway and co-workers in five cases, with four patients having pruritic skin while two having cutaneous skin ulcers. These cutaneous problems usually seem to develop during the first three weeks of treatment; and once controlled, they do not seem to recur as the lithium dosage is increased at a future date.[20] Carter first documented psoriasis as a cutaneous side effect and reported the aggravation of psoriasis upon lithium treatment.[21] The cutaneous side effects reported with lithium treatment include acneiform eruption, exfoliative dermatitis, pityriasis versicolor, pruritic maculopapular erythematous eruption, dermatitis herpetiformis and Darier's disease. Alopecia which is of the diffuse non-scarring type is seen in 12-19% of patients on long-term lithium treatment. In some cases, alopecia is related to lithium-induced hypothyroidism. The reported prevalence rate of such adverse effects varies from 3 to 45%. Acneiform eruptions, psoriasis, maculopapular eruptions and follicular eruptions are the commonest cutaneous reactions to lithium. How lithium brings about these reactions is still not fully understood. Lithium tends to aggravate cutaneous conditions that are associated with the pathological findings of neutrophilic infiltration. In addition to cutaneous effects, lithium causes an increase in circulating neutrophil level, an effect that would reverse within a week after termination of treatment. The mechanism is not well established but its action on cAMP is thought to be important. By reducing the level of cAMP, lithium enhances neutrophil chemotaxis and promotes lysosomal release from leukocytes; but whether it has additional effects such as alteration of adhesion molecule expression is not clear.[22] Hidradenitis suppurtiva related to lithium use may be accounted by neutrophilic chemotaxis and degranulation, which induce the inflammatory cascade (as in psoriasis). Follicular plugging due to direct influence of lithium on the follicular keratinocytes (as in acne) resulting in follicular occlusion adds to the pathology [Table 5].[23]

**Table 5:** Dermatologic effects of lithium

Dermatologic adverse effects – 3 to 45% acneiform eruption
Exfoliative dermatitis
Psoriasis
Pityriasis versicolor
Pruritic maculopapular erythematous eruption
Dermatitis herpetiformis
Darier's disease
Alopecia (diffuse non-scarring type) – 12 to 19%
Lithium used to treat seborrheic dermatitis, eczematoid dermatitis and genital herpes
Aggravates cutaneous conditions associated with neutrophilic infiltration
Lithium ↓ cAMP level and ↑ neutrophil chemotaxis and lysosomal release
Lithium causes follicular plugging and occlusion

The treatment strategies include alternative options to lithium, supportive measures and dermatological interventions directed to specific skin lesions.

## LITHIUM USE IN RESPIRATORY DISEASES

The inositol phospholipid-derived second messengers are involved in the initiation and maintenance of airway smooth muscle contraction. Lithium, through its effects on cell signal transduction and ion-transport pathways, would be likely to protect the airways against constrictor stimuli. A study has shown that lithium reduces bronchial reactivity in airway smooth muscle and is a possible agent for the treatment of asthma.[24] A double-blind placebo-controlled crossover study of lithium found that lithium had no advantage over placebo in the treatment of asthma.[25] There is also evidence for the development of asthma following cessation of lithium therapy. Therefore, careful monitoring of asthma control is advisable when discontinuing lithium carbonate.[26] The bronchodilators used in the treatment of asthma increase the excretion of lithium; so a higher dose is necessitated to maintain the therapeutic level of lithium in such patients.

Lithium use in chronic obstructive pulmonary disease may precipitate hypercapnia.[11] Lithium treatment is also reported to be associated with pulmonary hypertension. The mechanism by which lithium produces pulmonary hypertension is unclear. It is supposed to be due to the effect of lithium on serotonin system which is necessary for pulmonary vessel remodeling during pulmonary hypertension.[27]

## LITHIUM IN PREGNANCY AND LACTATION

Lithium is a USFDA pregnancy “category D” drug, implying that there is positive evidence for fetal risk with lithium, though the potential benefits may outweigh the risk in some cases. The incidence of major malformations in fetal life due to lithium exposure ranges from 4% to 12%, while the rate in unexposed infants ranges from 2% to 4%. The risk of Ebstein's anomaly exists especially if the drug is taken during weeks 2-6 post-conception.[28] The Register of Lithium Babies, a voluntary physician-reporting database, noted a 400-fold higher rate of cardiovascular malformations in offspring exposed *in utero* compared with the general population. Subsequent investigations identified a risk around 0.05-0.1% of Ebstein's anomaly among offspring of lithium users, which is 20 to 40 times higher than the rate in the general population. Thus, the relative risk for Ebstein's anomaly with prenatal lithium exposure is somewhat higher than in the general population, although the absolute risk remains small. Lithium-exposed infants were found to weigh significantly more than the comparison subjects.[29] Other types of lithium-related fetal and neonatal complications include premature delivery, floppy infant syndrome, transient neurodevelopmental deficits, nephrogenic diabetes insipidus, thyroid dysfunctions and rarely, polyhydramnios. However, the frequency of these remains unknown. Recently, a case of lithium-associated anencephaly also has been described. Additionally a higher lithium concentration in maternal serum at delivery is found to be associated with increased risk of perinatal complications. Recent reports conclude that the use of lithium during pregnancy is associated with no significant increase of congenital anomalies.[28]

The strategy for management of pregnant women on lithium varies; some authorities have suggested the maintenance of lithium treatment for bipolar women with severe forms of the disease. This is because the potential lithium-related teratogenicity in these cases is outweighed by the risks deriving from drug discontinuation and disease relapse. Others have recommended the following treatment plan: Stop lithium prior to conception, (b) restart the compound during trimester 2 or 3, (c) discontinue lithium prenatally and (d) restore the treatment postnatally. In any case, fetal cardiac ultrasonography is recommended at weeks 18 and 20 of gestation when the maternal clinical conditions require lithium therapy. Lithium serum levels, which may be affected by vomiting, sodium intake and febrile illnesses, should be closely monitored. The increase of renal lithium excretion during pregnancy may require an increase of the lithium dosage, whereas the drug dosage should be decreased at the beginning of labor, to reduce the risk of toxicity related to the abrupt reduction of vascular volume postparturition. In case of prolonged labor, adequate hydration of the mother should also be maintained.[28]

Lithium postpartum prophylaxis has been found to reduce the rate of relapse from near 50% to less than 10%. A recent study shows that serum lithium concentrations are substantially lower in nursing infants than previous estimates. Lithium concentrations in infant serum (0.16 mEq/liter), breast milk (0.35 mEq/liter) and maternal serum (0.76 mEq/liter) followed an approximate “rule of halves.” Breast milk contained about half the concentration of maternal serum, and infant serum had about half the level in breast milk, so that infant serum contained about one quarter the concentration of lithium in maternal serum.[30] The diminished renal clearance in neonates can elevate serum levels of lithium. The major concern regarding appreciable lithium levels is the propensity for rapid dehydration in neonates with febrile illnesses. Another consideration is that the longer-term effects on the infant of sustained lithium levels are not known.[29] Not many reports have described detrimental effects in newborns whose mothers continued to take lithium during the postpartum period. The reported effects include lethargy, hypothermia, hypotonia and T-wave modifications on ECG. High concentrations of the drug were reported in infant serum, breast milk and maternal serum, with ranges of 5–200% both in infant serum and breast milk and of 24-72% in maternal serum in these studies.[24] The American Academy of Pediatrics (AAP) has stated that lithium has been associated with significant effects on some nursing infants and recommends that breast-feeding be undertaken with caution by women undergoing lithium treatment. In a breast-fed infant exposed to lithium, lithium serum concentrations and the complete blood count (CBC) should be monitored [Table 6].[29]

**Table 6:** Lithium in pregnancy and lactation

Category D drug
No significant increase of congenital anomalies
Incidence of major malformations – 4% to 12%
Ebstein's anomaly risk 20 to 40 times the risk in general population
Ebstein's anomaly – 0.05% to 0.1%
Premature delivery
Floppy infant syndrome
Transient neurodevelopmental deficits
NDI
Thyroid dysfunctions
Polyhydramnios (rare)
Infant serum one quarter the concentration of lithium in maternal serum
Not many reports of detrimental effects in newborns
Reports of lethargy, hypothermia, hypotonia and T-wave modifications
AAP recommendation – breast-feeding with caution
Serum lithium and CBC monitoring of infant

Although lithium is secreted through milk, there is no deleterious effect described. The chances of any organ injury in the neonate are rather remote. No neurobehavioral sequelae have been described in infants who have been exposed to lithium. The mother has to make her own choice, along with the support of her husband and the treating physician, whether the gain from breast-feeding outweighs the losses when breast-feeding is avoided.

## LITHIUM USE IN ELDERLY

Elderly individuals require lower doses of lithium to achieve similar serum concentrations as those in younger adults. A study on the use of lithium in elderly has shown older patients (aged 70–79 years) required a dose 31% lower than those aged <50 years.[31] Bioavailability of lithium is not expected to be altered by increasing age as lithium is not subject to first-pass metabolism.[32] Lithium distribution in elderly is influenced by physiologic change related to body composition, particularly total body water. There is a decrease in total body water with advancing age, which results in a lower volume of water per kilogram of body weight. So the same dose of lithium in an older person would have less water for the lithium to distribute into, resulting in a higher serum lithium concentration.[33] Dehydration in the elderly due to age-related deficits in thirst and water intake regulation also increase the serum level of lithium.[34] The decline of glomerular filtration rate (GFR) with increasing age results in a decrease in lithium clearance and increased serum level.[32] The drugs commonly used in the elderly, like diuretics, ACE inhibitors, calcium antagonists, nonsteroidal anti-inflammatory drugs (NSAIDs) and psychotropic medications, alter the serum levels of lithium. There is also a difference in lithium tolerability with age, and the prevalence of hand tremor with lithium increases with age.[33] In the elderly, neurotoxicity clearly occurs at serum lithium levels which are considered “therapeutic” in general adult populations.[35]

There are no placebo-controlled randomized trials of lithium in old age, and recommendations for clinical use are based on extrapolations from pharmacokinetic studies, anecdotal reports and clinical experience in geriatric psychiatry. There is agreement, however, that the dosage and serum concentrations of lithium need to be much reduced in the elderly population, particularly so in the very old and frail elderly. Guidelines for serum lithium concentrations are based on limited evidence; and a recent study recommends a low mean serum lithium concentration (approximately 0.5 mmol/L), which may be achieved using a mean dose of just over 400 mg/day in a single-dose regimen.[36] The dosage recommended amongst patients aged between 65 and 75 years ranges from 300 to 600 mg/day and rarely exceeds 900 mg/day. For patients aged more than 80 years or frail elderly, the dosage should range from 150 to 300 mg/day and should rarely exceed 450 mg/day.[32]

## LITHIUM USE IN CHILDREN AND ADOLESCENTS

Lithium is the most widely studied agent in the acute monotherapy for mania in children and adolescents. Although it is currently the only medication approved by the U.S. Food and Drug Administration (FDA) for the treatment of mania in children aged 12 years and above, this indication was based on results of adult studies rather than specific clinical trials performed in adolescents. Lithium monotherapy may be reasonably safe and effective for the treatment of acute mixed states in children and adolescents. Studies also show that lithium may be effective and safe for the treatment of the depressed phase of illness in adolescents with bipolar disorder.[37] At present, lithium treatment cannot be recommended for children under 12 years of age - except under inpatient conditions. The dosage and serum levels of lithium, as well as its adverse effects, are comparable with those known from adults.[37] It is recommended that the serum concentrations of lithium should be between 0.6 and 1.2 mmol/L.[38] Side effects have to be monitored very carefully. Serum concentrations higher than 1.5 mmol/L may pose problems [Table 7].[39]

**Table 7:** Lithium use in elderly and adolescents

Elderly individuals at lower doses of lithium to attain adult serum concentrations
Bioavailability of lithium not altered by increasing age
Elderly have ↓ volume of distribution and ↓ GFR; this ↑ S. Li levels
Higher incidence of neurotoxicity in elderly
65 to 75 years – dose 300 to 600 mg/day; maximum 900 mg/day >80 years or frail elderly – 150 to 300 mg/day and rarely exceed 450 mg/day
Cannot be recommended for children under 12 years of age
Adolescents dosage and serum levels comparable with those of adults

## LITHIUM TOXICITY

Lithium is minimally protein bound and has an apparent volume of distribution of 0.6 L/kg. The therapeutic dose is 300–2700 mg/d with desired serum levels of 0.7-1.2 mEq/L.[1] The plasma elimination half-life of a single dose of lithium is from 12 to 27 hours (varies with age) and increases to approximately 36 hours in elderly persons. Toxicity associated with lithium treatment is prevalent, and 75-90% of patients treated with lithium have symptoms and signs of toxicity at some point during their treatment. Many minor side effects may occur at serum levels of 0.6-1.2 mEq/L. Symptoms and signs of mild intoxication include tremor, nausea, diarrhea, blurred vision, vertigo, confusion and increased deep tendon reflexes. With levels >2.5 mEq/L, patients may experience more severe neurological complications such as seizures, coma, cardiac dysrrhythmia and permanent neurological impairment (often cerebellar).[40] Patients with preexisting EEG abnormalities, seizures and/or cerebral impairment may be at increased risk for acute neurotoxicity.[41] Around 15% are rated moderate-to-severe toxicity, but mortality is less than 1%.[42]

There are two types of lithium intoxications: acute and chronic. Acute lithium intoxication occurs when the patient ingests it as a suicide attempt or overdoses accidentally. Chronic lithium intoxication occurs when the patient's lithium dosage has been increased or when their renal function has been impaired, resulting in an increase in serum lithium levels. Other factors that might increase the risk of chronic lithium intoxication in previously stable patients include drug-drug interactions, concurrent illness resulting in decreased circulating volume and alternations in electrolyte concentrations (especially potassium, calcium and sodium). The magnitude of the serum lithium level and the duration of exposure to a high level of lithium are both correlated with risk of adverse effects.[41] The correlation between serum lithium level and intoxication is debatable, and serum lithium may correlate closely to severity of toxicity, at least in chronic poisoning; but most accept that the relationship is not close and that lithium levels have a very limited role in the assessment of a poisoned patient.[40]

Concomitant use of diuretics, angiotensin-converting enzyme inhibitors, calcium channel antagonists or nonsteroidal anti-inflammatory drugs has been associated with lithium toxicity through pharmacokinetic interactions. In general, documented interactions between lithium and psychotropic medications are usually attributed to pharmacodynamic mechanisms. A wide variety of antipsychotic drugs has been implicated in increased lithium toxicity, including haloperidol, thioridazine, chlorpromazine, clozapine and risperidone. It is hypothesized that neuroleptic drugs, phenothiazines in particular, might increase lithium influx in red blood cells and that the enhanced levels of lithium in the tissue may possibly be responsible for the neurotoxic effects. But the neurotoxic reaction between lithium and any antipsychotic drugs is a rare and mostly reversible event. Other drugs, such as carbamazepine, valproic acid, propranolol, have also been reported to increase the risk of lithium toxicity.[42]

Lithium intoxication remains a serious medical problem. If a patient shows signs of toxicity, stop lithium immediately, assess the serum lithium levels and also do a creatinine estimation and urinalysis. In case of lithium over-dosage, gastric lavage may be useful early after an acute overdose – to remove any remaining pills in the stomach. Whole bowel irrigation (WBI) with a polyethylene glycol electrolyte solution, at 1500 to 2000 cc/hour; or use of polystyrene sulfonate (SPS), a cation exchange resin, should be considered for adult patients with acute toxic ingestions of lithium, especially if lithium concentrations are rising. The use of sodium polystyrene sulfonate (SPS) is however impractical for because the equivalent dose of SPS is too high and would result in hypokalemia. Most patients with lithium intoxication are volume depleted and may require intravenous rehydration. Forced saline diuresis would theoretically increase lithium elimination by increasing glomerular filtration, but this has not been documented clinically. Urinary alkalinization has little effect on serum lithium concentrations. Sodium bicarbonate is not recommended because of the risk of hypokalemia and fluid overload.[43]

Hemodialysis is the cornerstone of therapy and should be considered early in treatment, when serum lithium levels are elevated, regardless of symptoms. Guidelines recommend that the following patients receive hemodialysis: those whose lithium levels exceed 6 mEq/L; those receiving long-term lithium therapy whose lithium levels exceed 4 mEq/L; those with severe neurologic symptoms, renal insufficiency or unstable hemodynamic status with lithium levels ranging from 2.5 to 4.0 mEq/L; and those with end-stage renal disease or an increasing lithium level after hospital admission and whose levels range from 1.0 to 2.5 mEq/L. The goal of dialysis is a lithium level below 1 mEq/L 6–8 hours after hemodialysis; and as levels often rebound, dialysis may need to be prolonged and/ or repeated [Table 8].[43]

**Table 8:** Lithium toxicity

75 to 90% symptoms and signs of toxicity at some point during lithium treatment
Mild intoxication – tremor, nausea, diarrhea, blurred vision, vertigo, confusion and increased deep tendon reflexes
>2.5 mEq/L – seizures, coma, cardiac dysrrhythmia and permanent neurological impairment (often cerebellar)
Preexisting EEG abnormalities, seizures, cerebral impairment ↑ acute neurotoxicity risk
Mortality less than 1%
No strong correlation between serum lithium level and intoxication
Diuretics, ACE inhibitors, CCBs, NSAIDs – ↑ lithium toxicity
Haloperidol, thioridazine, chlorpromazine, clozapine, risperidone – ↑ lithium toxicity
Treatment by gastric lavage, whole bowel irrigation with polyethylene glycol, rehydration, hemodialysis

## CONCLUSION

Adequate care has to be taken while using lithium, the “gold standard” mood stabilizer, in the medically ill. The use of lithium in patients with cardiovascular, renal, endocrine, pulmonary and dermatological comorbidity is reviewed here to guide the clinician for better patient management. Use of lithium during pregnancy and lactation and in pediatric and elderly population and essentials about the toxicity of lithium are also covered in this paper. The relative safety of lithium during breast-feeding and the “lithium-related nephrotoxic scare” are briefly outlined.
